# Case of Dislocation of the Eye

**Published:** 1853-03

**Authors:** William Jameson

**Affiliations:** Surgeon to Mercer’s Hospital


*Case of Dislocation of the Eye.* By WILLIAM JAMESON, M. D., F. R. S., Surgeon to Mercer's Hospital.—Dr. Jameson detailed before the Surgical Society of Ireland, Dec. 4th, 1852, a case of dislocation of the eye which had lately come under his observation in Mercer's Hospital :—Peter Nowlan, ætat. 30, a powerfully able and muscular man, a corn porter, was admitted into Mercer's Hospital on the 3d of November, at half past twelve at night. His wife informed me that he came home that evening at ten o'clock in a most intoxicated condition, and while staggering about his room, struck his right eye against a small iron hook or nail that was in a dresser, which entered at the outer angle of the upper eyelid of that side, and when she went to his assistance discovered his eye protruded from its socket. She was most anxious to remove him at once to hospital, but could not succeed in prevailing on him to go until half past twelve at night, when in a few minutes after this I saw him.

He was very boisterous and unruly, had a large check apron held close up to his eye, which he kept constantly rubbing and pressing against it. On its being removed, he presented a most peculiar, and I might add, frightful appearance. There was the right eye protruded out of the orbit, firmly fixed and immoveable, staring, elastic to the touch, and devoid of all power of vision. The cornea was dry, cloudy, and rather opaque, pupil moderately contracted, and uninfluenced by the light of a candle. There was no extravasation of blood, nor was there any vascularity of the conjunctiva, although its reflection from the upper lid on the globe of the eye was partially torn through. The inferior margin of the upper lid was not visible, as it was placed behind the globe and spasmodically closed.

With difficulty I could get him restrained, as he was such a powerful man, but having accomplished it, I then, with two fingers of my left hand, elevated the upper lid, at the same time, with the finger and thumb of my right, pressed the ball of the eye, and immediately it was drawn back with a distinct snap, and the lids closed over its anterior surface. I now, for the first time, observed the small wound before alluded to at the outer angle of the upper lid, but could not ascertain or form any conjecture at the time what amount of injury he might otherwise have sustained. I therefore had him conveyed to bed, and ordered cold to be assiduously applied to the part for the remainder of the night.

4th. The following morning, at visiting hour, we found him sober, but recollecting little of what had occurred. His eyelids were a little swollen; there was some slight vascularity of the conjunctiva; the *cornea was clear*, *shining*, and moist, and the tears ran down the cheek; he could distinguish the daylight; complained of pain in the head, and a deep pain in the globe of the eye, with full pulse. He was ordered to have ℥xvi. of blood taken from his arm, bowels to be freely opened, and the cold to be continued to the part.

5th. Lids less tumid; pain and vascularity of conjunctiva almost gone; complains of the sensation as if gravel were between the lids; vision improved, but sees objects imperfectly, as through a thick haze. Ordered the tart. ant. mist., low diet, and the cold application to be continued.

6th. All pain gone; conjunctival vascularity less; sensation as if gravel were beneath the lids gone; vision nearly restored; has complete power over all the motions of the eye. Continue all.

7th. Convalescent; no suffusion; no pain; vision complete. 9th. Discharged cured.

The foregoing case Dr. Jameson considered to be one of great interest, when we reflect on the novelty and nature of the accident, and the mode of its being inflicted. In the first instance, the great escape the orbital plate of the frontal bone had of being pierced, and consequent injury to the anterior lobe of the brain. Again, the length of time the cornea was left uncovered by the palpebræ, being two hours and a half, and all that time coarsely rubbed by the apron. The great state of tension the optic nerve must have been kept in without permanent loss of vision. The escape the muscular attachments had of being torn from their origins, which evidently must have been the case from the subsequent perfect control retained over all the motions of the eye, as soon as the very slight amount of inflammation produced by the accident was removed. The powerful contraction of the orbicularis muscle behind the globe, with the complete restoration of vision. And finally, the trifling amount of constitutional disturbance and local inflammation that followed what appeared to be at first sight so very grave an accident to so very delicate an organ. These, he said, are points which add considerably to the interest of the case.

Dr. Jacob said the Society were greatly indebted to Dr. Jameson for the very interesting case he had just brought under their notice. As far as he could recollect, one of exactly the same kind was scarcely to be found on record. He thought it was a case which ought not to be dismissed from their consideration, without some suggestion to account for the occurrence of such an accident. He would solve the matter in this manner: Some persons were born with very large eyes and shallow orbits, and often, while examining the eyes of such persons, he found that by pressing the lids above and below, he could with ease get a view of the back of the eye. It was not that he merely saw one-half of the eye, but by a little manipulation of the eyelids, in persons with a shallow orbit and a large eyeball, he could obtain a view of the posterior part of the ball. Now, if by means of violence, the lids were tucked in above and below, they would grip the back of the eye and produce a downright protrusion of the organ from the orbit. He could not conceive any other way in which the accident could happen, because, as they might recollect, neither the muscles nor the optic nerve were torn in the case described by Dr. Jameson.

Dr. Jameson—The circumstance of the eye being drawn back with a distinct snap, shows that the muscles were at least on the stretch.

Dr. Jacobs—Yes, that is quite evident.—*Dublin Med. Press.*

